# Supplementary figures and images for: Grape polyphenols reduce gut-localized reactive oxygen species associated with the development of metabolic syndrome in mice
Source: PLoS One. 2018 Oct 11;13(10):e0198716. doi: 10.1371/journal.pone.0198716 (PMC6181265; doi:10.1371/journal.pone.0198716)

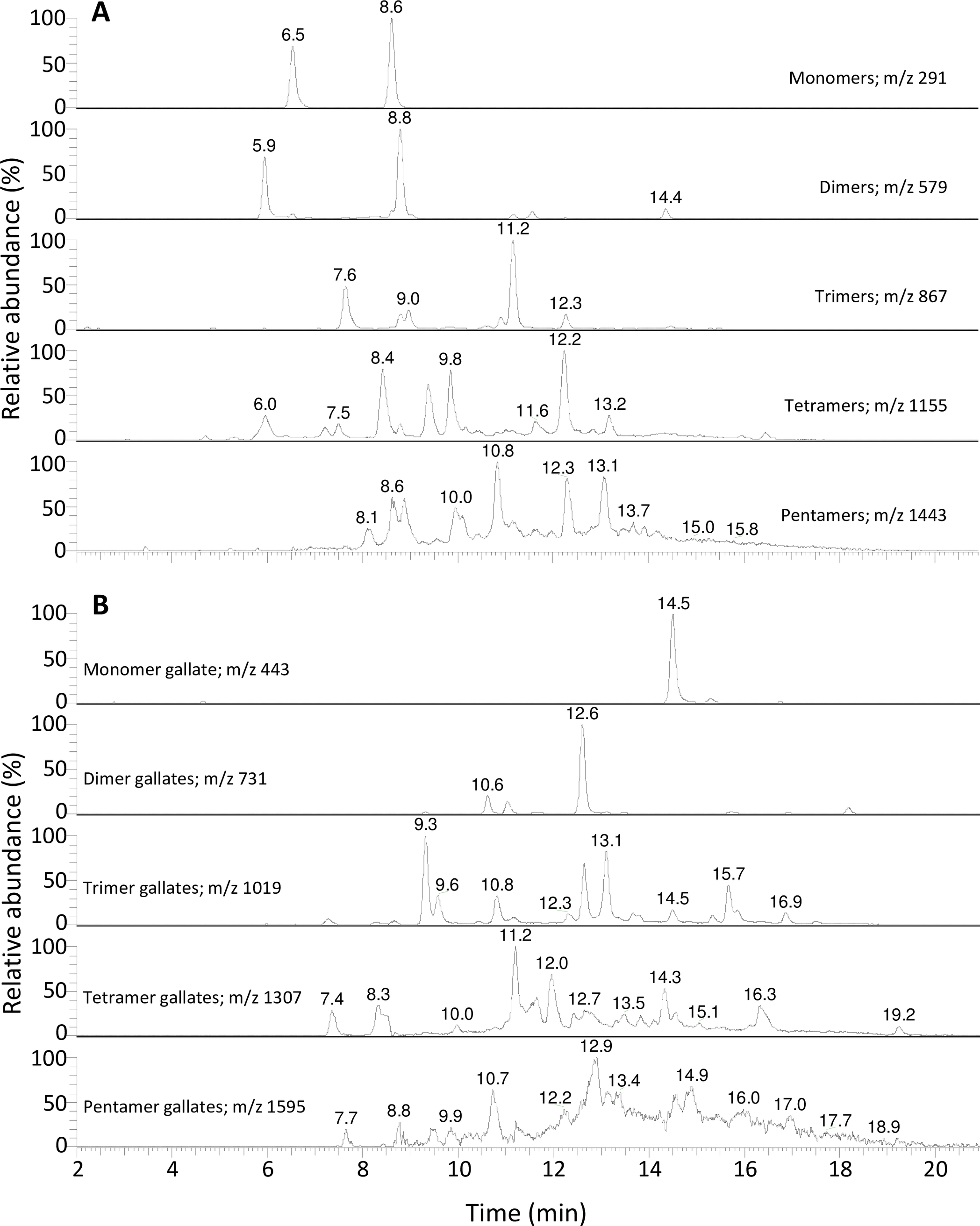

Supplement: S1 Fig — A–oligomeric PACs; B–oligomeric PAC monogallates. (TIF) [file pone.0198716.s001.tif]
